# Pharmacogenetic Variability and Quality of Life in Adolescent Patients with Schizophrenia: The Impact of Metabolizer Status, Symptom Severity, and Adverse Reactions to Antipsychotic Treatment

**DOI:** 10.3390/jcm15082912

**Published:** 2026-04-11

**Authors:** Bianca Oana Bucatos, Ana-Maria Romosan, Liana Dehelean, Radu Ștefan Romosan, Adriana Cojocaru, Nilima Rajpal Kundnani, Abhinav Sharma, Delia Mira Berceanu Vaduva, Laura Alexandra Nussbaum

**Affiliations:** 1 Department of Neurosciences-Psychiatry, “Victor Babes” University of Medicine and Pharmacy, 3000417 Timisoara, Romania; bianca.bucatos@umft.com (B.O.B.); lianadeh@umft.ro (L.D.); romosan.radu@umft.ro (R.Ș.R.); 2 PhD School Department, Victor Babes University of Medicine and Pharmacy, 300041 Timisoara, Romania; 3 Centre for Cognitive Research in Neuropsychiatric Pathology, “Victor Babes” University of Medicine and Pharmacy, 300041 Timisoara, Romania; 4 Center for Studies in Preventive Medicine, “Victor Babes” University of Medicine and Pharmacy, 300041 Timisoara, Romania; 5 Department of Neurosciences-Pedopsychiatry, “Victor Babes” University of Medicine and Pharmacy, 3000417 Timisoara, Romanianussbaum.laura@umft.ro (L.A.N.); 6 University Clinic of Internal Medicine and Ambulatory Care, Prevention and Cardiovascular Recovery, Department VI-Cardiology, “Victor Babes” University of Medicine and Pharmacy, 300041 Timisoara, Romania; 7 Research Centre of Timisoara, Institute of Cardiovascular Diseases, “Victor Babes” University of Medicine and Pharmacy, 300041 Timisoara, Romania; 8 Department XIV—Microbiology, “Victor Babeș” University of Medicine and Pharmacy, 300041 Timisoara, Romania

**Keywords:** schizophrenia, adolescents, CYP2D6, pharmacogenetics, quality of life, antipsychotic adverse effects, hyperprolactinemia, extrapyramidal symptoms, PQ-LES-Q, PANSS

## Abstract

**Background**: Schizophrenia in adolescence disrupts neurodevelopment and long-term functioning. While symptom reduction remains a primary treatment goal, quality of life (QoL) represents a critical, patient-centered outcome. Pharmacogenetic variability, particularly in CYP2D6 metabolism of second-generation antipsychotics, may influence tolerability and subjective well-being beyond symptom control. **Materials and Methods**: Forty-seven adolescents (aged 14–18 years) diagnosed with schizophrenia (DSM-5) were followed in routine clinical care. CYP2D6 genotyping classified patients as normal metabolizers (NM, n = 27) or reduced-function metabolizers (RFM, including intermediate/poor, n = 20). Symptom severity was assessed with PANSS, QoL was assessed with the Pediatric Quality of Life Enjoyment and Satisfaction Questionnaire (PQ-LES-Q), and adverse effects (hyperprolactinemia, extrapyramidal symptoms, sedation, metabolic changes) were monitored. Non-parametric tests and multiple linear regression were applied. **Results**: At 12 months, RFM patients showed significantly higher PANSS scores, markedly more adverse reactions (95% vs. 48.1%), and lower PQ-LES-Q total and domain scores (all *p* < 0.0001) compared to NM patients. A regression analysis identified the metabolizer status (β = −0.410, *p* = 0.001), extrapyramidal symptoms (β = −0.248, *p* = 0.003), sedation (β = −0.193, *p* = 0.029), and hyperprolactinemia (β = −0.190, *p* = 0.012) as independent predictors of a reduced QoL, explaining 84% of the variance. The residual symptom severity was not independently associated. **Conclusions**: In adolescent schizophrenia, the CYP2D6-reduced metabolizer status is the strongest independent predictor of long-term QoL impairment, associated primarily through a substantially higher burden of treatment-related adverse effects (metabolic, endocrine, neurological, and sedative) rather than through persistence of psychotic symptoms alone. These findings support early pharmacogenetic testing to guide individualized dosing and improve tolerability and patient-reported outcomes.

## 1. Introduction

Schizophrenia is a chronic and disabling psychiatric disorder marked by disturbances in perception, cognition, affect, and behavior, most commonly emerging during late adolescence or early adulthood [1,2]. Although its lifetime prevalence is relatively low, estimated at approximately 0.4–0.7%, the disorder accounts for a disproportionate share of global disability and functional impairment [3,4]. Variations in incidence according to sex, urbanicity, and migration status reflect its complex and multifactorial etiology [5]. When schizophrenia manifests during adolescence, it disrupts neurodevelopment at a particularly vulnerable stage, interfering with academic progress, social integration, and identity formation.

Clinical research in schizophrenia has traditionally focused on symptom reduction. The Positive and Negative Syndrome Scale (PANSS), introduced by Kay et al. [6], remains a cornerstone for quantifying symptom severity. Subsequent work has clarified how changes in PANSS scores translate into clinically meaningful response categories [7].

Standardized remission criteria based on specific symptom thresholds were later proposed by Andreasen et al. [8]. These advances have strengthened the reliability and interpretability of clinical outcomes across trials and longitudinal studies.

Yet symptom remission does not necessarily mean recovery. Over the past two decades, quality of life (QoL) has become recognized as a distinct and essential outcome in schizophrenia research. Katschnig emphasized that QoL captures subjective well-being, life satisfaction, and functioning, dimensions not fully reflected in symptom ratings [9]. Awad and Voruganti similarly argued that treatment success must incorporate patient experience and perceived tolerability [10]. This distinction is especially important in adolescents, for whom daily functioning, peer relationships, autonomy, and future aspirations are central to development.

The Quality of Life Enjoyment and Satisfaction Questionnaire (Q-LES-Q) was developed to measure subjective satisfaction across life domains [11], and its pediatric adaptation (PQ-LES-Q) extends this framework to younger populations [12]. Both the long and abbreviated forms have demonstrated solid psychometric validity in psychiatric samples [13,14]. These instruments assess emotional well-being, social functioning, independence, cognitive and physical functioning, and leisure satisfaction—domains directly relevant to adolescents living with schizophrenia.

Second-generation antipsychotics represent the standard pharmacological treatment for youth with schizophrenia. Although generally associated with improved neurological tolerability compared with first-generation agents, they are not free from clinically significant adverse effects. Prospective data have shown that cardiometabolic abnormalities, including weight gain, dyslipidemia, and insulin resistance, may develop rapidly in children and adolescents after treatment initiation [15]. These changes are not merely laboratory findings; they can influence physical health, self-image, and long-term cardiovascular risk.

Hyperprolactinemia is another frequent adverse effect, particularly with risperidone and related agents [16,17,18,19]. An umbrella review synthesizing the available evidence confirms both the prevalence of this complication and the range of therapeutic approaches for its management [20]. For adolescents, prolactin-related hormonal changes may carry particular psychosocial weight, affecting sexual development, body image, and self-esteem.

Although less common than with first-generation antipsychotics, extrapyramidal symptoms (EPSs) and sedation remain clinically meaningful in youth treated with second-generation agents [21].

Beyond clinical factors, variability in the treatment response and adverse-effect burden is influenced by pharmacogenetic differences. The cytochrome P450 enzyme CYP2D6 plays a major role in the metabolism of several antipsychotics, including risperidone and aripiprazole [22,23,24]. Genetic polymorphisms result in distinct metabolizer phenotypes that substantially influence plasma drug concentrations at standard doses [23]. Systematic reviews in pediatric populations have shown that the CYP2D6 phenotype affects risperidone pharmacokinetics, prolactin elevation, weight changes, and extrapyramidal symptoms [25], and meta-analytic data confirm robust associations between the CYP2D6 functional status and risperidone exposure [26].

Clinical studies have further linked the CYP2D6 genotype to extrapyramidal side effects in schizophrenia [27,28,29], and regulatory labeling for aripiprazole explicitly acknowledges dose adjustments for poor metabolizers [30]. Despite strong evidence linking CYP2D6 variability to pharmacokinetics and discrete adverse events, comparatively limited research has examined its broader impact on patient-reported outcomes such as quality of life, particularly in adolescent clinical populations.

### Objectives

The present study aimed to explore the role of pharmacogenetic variability (anomalies related to CYP2D6 mutations) in shaping long-term quality of life in adolescent patients diagnosed with schizophrenia and receiving antipsychotic treatment. The main objectives were to assess quality of life (general and specific domains) comparatively between normal (extensive) metabolizers and patients with reduced metabolic function, and to examine the relationship between quality of life, symptom severity and adverse reactions and antipsychotic treatment in these patients.

The present study therefore examined the relationship between the CYP2D6 metabolizer status, the symptom severity measured by PANSS, treatment-related adverse effects, and quality of life assessed using PQ-LES-Q. By integrating pharmacogenetic, clinical, and patient-reported data, this study sought to clarify whether the metabolizer status functions as an upstream determinant of long-term subjective well-being.

## 2. Materials and Methods

This prospective observational study included 47 adolescents aged 14–18 years diagnosed with schizophrenia according to DSM-5 criteria [31], with reference to DSM-5-TR updates for diagnostic continuity [32], confirmed by a structured clinical interview and a baseline PANSS total score indicative of active illness. All had a clinical indication for treatment with a second-generation antipsychotic (risperidone, aripiprazole, or olanzapine). The exclusion criteria included active substance abuse (confirmed by urine drug screen), refusal of consent by the patient or guardian, or age outside the specified range. The antipsychotic selection, initial dosing, and any subsequent changes were determined solely by the attending physician according to clinical judgment, with no external protocol-driven interventions.

The symptom severity was assessed at baseline and at 12 months using the Positive and Negative Syndrome Scale (PANSS) [6]. Interpretation of symptom changes was informed by established response thresholds [7] and remission criteria [8]. Quality of life was evaluated using the Pediatric Quality of Life Enjoyment and Satisfaction Questionnaire (PQ-LES-Q) [12], derived from the original Q-LES-Q instrument [11], with psychometric validity supported by subsequent studies [13,14]. The PQ-LES-Q was administered only by trained professionals at the clinic.

Prior to treatment initiation and throughout the follow-up period, the patients underwent comprehensive clinical and paraclinical evaluations, including vital signs, anthropometric measurements (with BMI expressed as the percentage change relative to age- and sex-adjusted norms), and laboratory investigations (including a metabolic panel, prolactin, fasting glucose and insulin). Adverse effects were systematically monitored using the UKU Side Effect Rating Scale and the Barnes Akathisia Rating Scale, supplemented by a clinical interview. Extrapyramidal symptoms and sedation were recorded as present or absent and expressed as percentages within the cohort.

CYP2D6 genotyping was performed, and the participants were classified as normal metabolizers or reduced-function metabolizers, including intermediate and poor metabolizers, in accordance with established genotype–phenotype translation frameworks [23,24,30].

The adverse effects monitored during follow-up included hyperprolactinemia, extrapyramidal symptoms, sedation, a BMI increase greater than 7%, and fasting insulin levels above 25 µIU/mL. The monitoring approaches were consistent with established metabolic and endocrine risk literature in youth receiving antipsychotics [15,16,17,18,19,20].

The study was conducted at the Child and Adolescent Psychiatry Clinic in Timișoara, Romania. All procedures were approved by the hospital’s ethics committee, and written informed consent was obtained from the legal guardians of all participants prior to inclusion.

### Statistical Analysis

Statistical analyses were performed using IBM SPSS Statistics version 20. Normality of distribution was assessed using the Shapiro–Wilk test [33]. Given non-Gaussian distributions, non-parametric tests were applied, including the Wilcoxon signed-rank test for paired comparisons [34], the Mann–Whitney U test for independent comparisons [35], and Spearman’s rank correlation for associations [36]. Multiple ordinary least-squares regression was conducted to determine predictors of the PQ-LES-Q total score at 12 months. The residual independence was evaluated using the Durbin–Watson statistic [37], and multicollinearity was assessed in accordance with variance inflation factor guidance [38]. Reporting adhered to the STROBE recommendations for observational studies [39].

## 3. Results

As mentioned in the description of the statistical analysis, for the most part, the data did not follow a Gaussian distribution (*p* < 0.05). The results of both the Kolmogorov–Smirnov and Shapiro–Wilk tests can be seen in Table 1.

The study included 47 adolescent patients diagnosed with SCZ, with a mean age of 15.45 years (SD = 1.27); age range: 14–18 years.

During the 12-month period, in 24 (51.1%) patients, the antipsychotic medication needed to be changed, either due to an improper response (persistence of psychotic symptomatology) or the occurrence of adverse reactions. Certain patients required a combination of two atypical antipsychotics. Throughout the study, the choice of antipsychotic medication was left entirely to the attending physician, who established the necessary dosage and changes in medication when needed, with no exterior intervention whatsoever. Therefore, in this study, we only refer to the current antipsychotic medication.

Because the sample contained only two intermediate metabolizers, for the statistical analysis, the metabolizer status was divided into two categories: NM—normal (extensive metabolizers) and RFM—reduced-function metabolizers, which contained both intermediate metabolizers (IM) and poor metabolizers (PM).

The socio-demographics and clinical characteristics are presented in Table 2.

Table 2 and T0 values regard the: BMI values (Z = −4.86, *p* < 0.0001), fasting insulin levels (Z = −5.88, *p* < 0.0001), prolactin levels (Z = −4.71, *p* < 0.0001), PANSS total (Z = −5.97, *p* < 0.0001) and PQ-LES-Q total (Z = −5.16, *p* < 0.0001). The BMI values, fasting insulin levels, prolactin levels and PQ-LES-Q scores increased significantly from baseline to the 12-month follow-up, while the PANSS total scores registered a significant decrease.

### 3.1. Quality of Life, Metabolizer Status and Adverse Reactions to Antipsychotic Medication

Adverse reactions to antipsychotic medication were significantly more frequent in the RFM group than in the NM group (χ^2^ = 11.61, *p* = 0.001), with 13/27 (48.1%) NM and 19/20 (95%) RFM patients presenting with adverse effects.

The current antipsychotic treatment, the most frequent adverse reactions found in these patients, and the PANSS total scores and PQ-LES-Q scores (baseline and at 12 months) for NM and RFM patients are presented in Table 3. For PQ-LES-Q, in addition to the total scores, and based on the existing literature, we calculated sub-scores for five domains, as follows: GEWB (general and emotional well-being), SF (social functioning), IDL (independence and daily life), CPF (cognitive and physical functioning), and LLS (leisure and life satisfaction).

Adverse reactions to antipsychotic treatment such as an increase in BMI over 7%, hyperprolactinemia, sedation, extrapyramidal symptoms and sex hormone imbalances (dis-/amenorrhea, gynecomastia, galactorrhea) were significantly more frequent in the RFM patient group (Table 3).

Also revealed in Table 3, at baseline, there were no significant differences between NM and RFM patients regarding the PANSS total scores and PQ-LES-Q scores (total and specific-domain sub-scores). In contrast, the PANSS total scores at the 12-month follow-up were significantly higher in the RFM group compared to the NM group, while the PQ-LES-Q total scores and the specific domain sub-scores were significantly lower in the RFM group.

At the 12-month follow-up, the PQ-LES-Q total scores correlated negatively with the PANSS total scores (Spearman’s rho [r] = −0.66, *p* < 0.0001) and with the insulin levels (Spearman’s rho [r] = −0.49, *p* < 0.0001). The GEWB sub-scores also correlated negatively with the PANSS total scores (Spearman’s rho [r] = −0.72, *p* < 0.0001), with the insulin levels (Spearman’s rho [r] = −0.49, *p* < 0.0001) and with the BMI values (Spearman’s rho [r] = −0.34, *p* = 0.02). The SF sub-scores correlated negatively with the PANSS total scores (Spearman’s rho [r] = −0.59, *p* < 0.0001) and with the insulin levels (Spearman’s rho [r] = −0.33, *p* = 0.02). These two negative correlations were also significant with regards to the IDL sub-scores (with PANSS total scores: Spearman’s rho [r] = −0.53, *p* < 0.0001; with insulin levels: Spearman’s rho [r] = −0.39, *p* = 0.006), CPF (with PANSS total scores: Spearman’s rho [r] = −0.52, *p* < 0.0001; with insulin levels: Spearman’s rho [r] = −0.44, *p* = 0.002), and LLS (with PANSS total scores: Spearman’s rho [r] = −0.56, *p* < 0.0001; with insulin levels: Spearman’s rho [r] = −0.42, *p* = 0.003).

As revealed in Table 4, quality of life (general scores and specific domain sub-scores) was, for the most part, significantly reduced in patients presenting adverse effects to the antipsychotic medication.

### 3.2. Predictive Factors for Quality of Life in Adolescent Patients Diagnosed with SCZ

In order to find factors with predictive value on the quality of life in adolescent remitted patients with SCZ that were genetically assessed for CYP2D6 mutations, a multiple OLS regression analysis was computed. The regression tested if symptomatology at the 12-month follow-up, the metabolizer status and the presence of several adverse reactions to antipsychotic treatment (hyperprolactinemia, sedation, fasting insulin levels > 25 µIU/mL, BMI increase over 7%, and extrapyramidal symptoms) could significantly predict quality of life in these patients (PQ-LES-Q total scores at T12). The model was statistically significant: F(7) = 29.22, *p* < 0.0001. It explained 84% of the variance in the PQ-LES-Q total scores. The Durbin–Watson test yielded a result of 1.46 and the VIF coefficients ranged from 1.18 to 2.89, while the tolerance coefficients were all above 0.2, ranging from 0.34 to 0.84 (no significant multicollinearity detected). After adjusting for all the variables included in the model, the metabolizer status (B = −7.99, *p* = 0.001), extrapyramidal symptoms (B = −5.47, *p* = 0.003), sedation (B = −4.73, *p* = 0.029), and hyperprolactinemia (B = −4.47, *p* = 0.012) represented factors that were significantly associated with lower PQ-LES-Q scores at 12 months. Although an increase over 7% in BMI from baseline to one year showed a tendency towards a negative association with quality of life (B = −2.97, *p* = 0.07), it was not statistically significant. The PANSS total scores (B = −0.04, *p* = 0.613) and fasting insulin levels >25 µIU/mL (B = −2.77, *p* = 0.10) were not significantly associated with the PQ-LES-Q scores (Table 5). Amongst the predictors, metabolizer status showed the strongest association with a lower quality of life (the largest standardized effect: β = −0.410), followed by extrapyramidal symptoms (β = −0.248), sedation (β = −0.193) and hyperprolactinemia (β = −0.190).

## 4. Discussion

This study examined how pharmacogenetic variability, symptom severity, and treatment-related adverse effects interact to shape long-term quality of life in adolescents with schizophrenia. The findings suggest that subjective well-being in this population is influenced more strongly by pharmacogenetic and tolerability factors than by symptom severity alone. This is consistent with a recent systematic review in pediatric populations, which found that CYP2D6 poor metabolizers have significantly higher risperidone concentrations and greater adverse effects (e.g., extrapyramidal and metabolic effects) compared to normal metabolizers [21].

Although the PANSS scores improved during follow-up, the symptom severity at 12 months was not independently associated with the PQ-LES-Q total score in a multivariate analysis. This result reinforces conceptual distinctions between clinical remission and subjective recovery. While reductions in PANSS scores correspond to clinically meaningful improvement [7] and remission criteria have been clearly defined [8], quality of life captures broader aspects of lived experience that extend beyond psychotic symptoms [9,10]. In adolescence, when social belonging and identity development are particularly salient, subjective experience may carry even greater weight than residual symptom levels [9,10].

Treatment-related adverse effects emerged as key determinants of a reduced quality of life. Hyperprolactinemia was independently associated with lower PQ-LES-Q scores. Pediatric studies have consistently documented prolactin elevation under atypical antipsychotics [16,17], and mechanistic reviews detail the endocrine and reproductive consequences of sustained hyperprolactinemia [18,19]. The umbrella review by Jiang et al. underscores both the frequency and clinical relevance of this complication [20]. For adolescents, prolactin-related hormonal disturbances may extend beyond laboratory abnormalities, influencing sexual maturation, body image, emotional regulation, and self-esteem, domains directly assessed within PQ-LES-Q constructs.

Extrapyramidal symptoms were also independently associated with a reduced QoL. Pharmacogenetic research has linked the CYP2D6 genotype to EPS vulnerability in schizophrenia [27,28,29], and controlled pharmacokinetic studies support genotype-dependent differences in risperidone exposure and neurological adverse effects [29]. Beyond their biological association with genotype, motor side effects can plausibly reduce functioning and treatment satisfaction, particularly in adolescents where visible symptoms may affect self-confidence, peer interaction, and participation in daily activities [27,28,29].

Sedation similarly predicted a diminished quality of life. Clinical trials in adolescents treated with second-generation antipsychotics document sedation as a frequent and sometimes dose-limiting adverse effect [21]. Although often characterized as manageable in adult populations, sedation in adolescents may interfere with school attendance, academic concentration, extracurricular engagement, and social participation. Given that PQ-LES-Q assesses domains such as cognitive and physical functioning, leisure satisfaction, and independence in daily life, sedation may exert a broad functional impact beyond symptom control alone.

The metabolic burden showed a trend toward association with a lower QoL, consistent with evidence that cardiometabolic changes emerge early during antipsychotic treatment in youth [15]. Although a BMI increase did not retain statistical significance in the fully adjusted model, weight gain and insulin dysregulation may nonetheless contribute cumulatively to psychosocial distress, reduced self-esteem, and concerns about physical health, particularly in adolescence, a developmental period characterized by a heightened sensitivity to body image and peer evaluation [15].

Approximately half of the participants required medication changes and received one of four different second-generation antipsychotics with varying degrees of CYP2D6 dependence. This real-world heterogeneity, while increasing ecological validity, represents a potential confounder. Future studies with larger samples may permit inclusion of medication type as a covariate.

The most striking finding in our study was that the CYP2D6 metabolizer status emerged as the strongest independent predictor of quality of life at 12 months, associated primarily through a higher burden of treatment-related adverse effects rather than through discrete side effects alone. Systematic reviews in pediatric populations have demonstrated that the CYP2D6 phenotype significantly influences risperidone pharmacokinetics and adverse-effect profiles [21,25], and meta-analytic evidence confirms robust associations between the CYP2D6 function and drug exposure [26]. While Li et al. (2022) demonstrated ethnic differences in CYP2D6 polymorphisms and metabolic activity among adult Han and Tibetan schizophrenia patients, the present study is, to our knowledge, the first to examine the longitudinal relationship between the CYP2D6 metabolizer status, treatment tolerability, and patient-reported quality of life in adolescents with schizophrenia [40]. Clinical studies have further linked the CYP2D6 genotype to extrapyramidal side effects [27,28,29], and regulatory guidance for aripiprazole includes dose adjustments for poor metabolizers [30].

The Dutch Pharmacogenetics Working Group (DPWG) now recommends reducing the dose of CYP2D6-metabolized antipsychotics, including risperidone and aripiprazole, for known poor metabolizers, reflecting the growing consensus that genotype-guided dosing may improve tolerability and safety [41].

Taken together, the available pharmacogenetic literature supports the interpretation that the CYP2D6 status may serve as a foundational determinant of the tolerability burden and, indirectly, patient-reported outcomes, rather than acting solely through discrete measurable adverse effects.

Importantly, the metabolizer status retained statistical significance even after adjusting for measured adverse effects. This suggests that pharmacogenetic variability may influence quality of life through mechanisms that extend beyond categorically defined side effects. Differences in the plasma drug concentration could produce subtle, but persistent, variations in subjective tolerability, cognitive clarity, affective stability, or energy levels that are not fully captured by standard adverse-effect checklists. Over time, such cumulative influences may shape broader perceptions of well-being and daily functioning.

The regression model explained a substantial proportion of the variance in the PQ-LES-Q scores, indicating that the combination of pharmacogenetic status and tolerability factors provides meaningful explanatory power. Symptom severity alone did not account for quality-of-life differences once these variables were considered. These findings align with recovery-oriented frameworks emphasizing that remission of psychotic symptoms does not necessarily equate to restoration of subjective well-being [9,10].

Adolescence represents a developmental stage in which medication side effects may carry amplified psychosocial consequences. Hormonal changes, weight gain, sedation, and movement abnormalities may influence peer relationships, academic identity, emerging autonomy, and self-concept. In this context, minimizing the adverse-effect burden may be as critical as achieving symptom remission. Incorporating CYP2D6 genotyping into clinical decision-making has the potential to inform dose optimization, enhance tolerability, and reduce exposure-related toxicity in vulnerable youth populations.

Moreover, recent survey data indicate that both patients and clinicians view pharmacogenetic testing for antipsychotic prescribing favorably, perceiving it as a strategy to reduce trial-and-error prescribing and improve therapeutic outcomes, further supporting the clinical relevance of integrating a pharmacogenetic assessment into psychiatric care [42].

This study has several strengths. It used a prospective 12-month design conducted under routine clinical practice conditions, increasing the real-world relevance. It integrated pharmacogenetic data (CYP2D6), symptom severity (PANSS), adverse effects, and quality of life (PQ-LES-Q), thereby moving beyond symptom reduction alone and aligning with recovery-oriented perspectives in schizophrenia care [9,10]. Importantly, the PQ-LES-Q was administered by a trained professional, which enhances the reliability and consistency of the assessments. The use of validated instruments and a multivariate regression model with strong explanatory power further supports the robustness of the findings. The focus on adolescents also addresses an underrepresented population in pharmacogenetic outcome research.

A primary limitation of this study is the relatively small sample size (N = 47) in relation to the number of predictors included in the multiple OLS regression model. According to some statistical heuristics, a larger participant-to-item ratio is often preferred to ensure maximum stability of the regression coefficients. The high degree of explained variance (R^2^ = 0.84; adjusted R^2^ = 0.81) suggests a very strong fit, which, in small clinical samples, carries an inherent risk of overfitting, which may limit the external validity of these findings to broader schizophrenia populations. However, despite the high R^2^, it is important to note that the gap between the R^2^ and the adjusted R^2^ was minimal (0.03), and all multicollinearity diagnostics (VIF, Durbin–Watson, tolerance) were well within acceptable ranges. This suggests that the predictors (especially the metabolizer status and extrapyramidal symptoms) provide a distinct and robust explanatory power. Furthermore, the high F-statistic and the significance levels of the individual coefficients (*p* < 0.0001) indicate that the effect size in this adolescent sample was large enough to be detected reliably, even with the current sample size. The high variance explained may also be a reflection of the unique homogeneity of the cohort (remitted adolescent patients). In this development stage, the subjective quality of life may be more sensitive to the physiological side effects of medication and the genetic metabolic efficiency than in other population groups (adults), where physiological factors often introduce more “unexplained” variance. Nevertheless, these results should be considered exploratory and require replication in larger, independent cohorts to confirm the exact magnitude of these predictive associations.

Because only two participants were intermediate metabolizers, they were grouped with poor metabolizers into a single “RFM” category. Although this grouping is consistent with common pharmacogenetic practice for small samples, it may have reduced our ability to detect phenotype-specific effects.

Although the present study focused on CYP2D6, several antipsychotics are also metabolized by CYP3A4 and CYP1A2, and pharmacodynamic variants may further influence the outcomes. CYP2D6 should therefore be viewed as one important component within a broader pharmacogenetic landscape.

Future research should prospectively evaluate genotype-guided antipsychotic treatment strategies in adolescent populations. Such approaches may clarify whether pharmacogenetic-informed prescribing can meaningfully improve the tolerability, adherence, and, ultimately, long-term quality of life.

## 5. Conclusions

In adolescents with schizophrenia, long-term quality of life appears to be associated more strongly with pharmacogenetic variability and treatment-related adverse effects than with symptom severity alone. The CYP2D6-reduced metabolizer status was the strongest independent predictor of a diminished quality of life at 12 months, even after adjustment for extrapyramidal symptoms, hyperprolactinemia, sedation, and metabolic changes. The symptom severity, as measured by PANSS, did not independently predict quality of life once these variables were considered.

These findings suggest that, in adolescent populations, subjective well-being may be shaped less by residual psychotic symptoms and more by the cumulative burden of treatment-related effects and pharmacokinetic variability. While symptom control remains fundamental in schizophrenia management, optimizing tolerability appears equally critical in preserving daily functioning, autonomy, and overall life satisfaction during a developmental period marked by social and psychological vulnerability.

The strong independent association of the CYP2D6 metabolizer status with quality of life underscores the potential value of integrating a pharmacogenetic assessment into individualized treatment planning. Identifying reduced metabolizers early in the course of treatment may allow for more precise dose adjustments, closer monitoring, and potentially reduced exposure-related toxicity. In turn, this approach may mitigate the adverse-effect burden and improve long-term patient-reported outcomes.

## Figures and Tables

**Table 1:** Results of the normality tests.

Tests of Normality						
	Kolmogorov–Smirnov ^a^			Shapiro–Wilk		
	Statistic	df	Sig.	Statistic	df	Sig.
Age	0.193	47	0.000	0.878	47	0.000
BMI baseline	0.187	47	0.000	0.906	47	0.001
BMI at 12 months	0.117	47	0.120	0.944	47	0.026
Prolactin level baseline	0.223	47	0.000	0.706	47	0.000
Insulin level baseline	0.116	47	0.129	0.961	47	0.114
Prolactin level at 12 months	0.279	47	0.000	0.657	47	0.000
Insulin level at 12 months	0.198	47	0.000	0.666	47	0.000
PANSS total baseline	0.106	47	0.200 *	0.957	47	0.078
PANSS total at 12 months	0.124	47	0.067	0.928	47	0.006
General and emotional well-being	0.159	47	0.004	0.949	47	0.040
Social functioning	0.296	47	0.000	0.849	47	0.000
Cognitive and physical functioning	0.216	47	0.000	0.918	47	0.003
Leisure and life satisfaction	0.303	47	0.000	0.852	47	0.000
Independence and daily life	0.156	47	0.006	0.931	47	0.008
PQ-LES-Q total score	0.193	47	0.000	0.937	47	0.014

* This is a lower bound of the true significance. ^a^ Lilliefors significance correction.

**Table 2:** Demographic, clinical, and pharmacogenetic characteristics of the study sample.

Sample Characteristics	Number of Patients (N = 47)		Percentage (%)	
Gender				
Males	23		48.9	
Females	24		51.1	
Genotype				
WT	27		57.4	
WT/*4	16		34.1	
WT/*3	1		2.1	
WT/*41	1		2.1	
*4	2		4.3	
Metabolizer status				
NM	27		57.4	
RFM (IM/PM)	20		42.6	
Current antipsychotic medication				
Aripiprazole	16		34.1	
Risperidone	9		19.1	
Olanzapine	18		38.3	
Quetiapine	4		8.5	
Adverse reactions to antipsychotic medication (T0–T12)				
	no	yes	no	yes
BMI increase (>7%)	28	19	59.6	40.4
Fasting insulin levels > 25 µIU/mL	37	10	78.7	21.3
Hyperprolactinemia	37	10	78.7	21.3
Sedation	38	80.9	9	19.1
Extrapyramidal symptoms	35	12	74.5	25.5
Sex hormone imbalances (dis-/amenorrhea, gynecomastia, galactorrhea)	36	11	76.6	23.4
	Mean		Standard deviation (SD)	
Age (years)	15.45		1.27	
BMI (kg/m^2^)—T0 baseline	23.83		5.47	
BMI (kg/m^2^)—T12	25.92		5.55	
Fasting insulin levels (µIU/mL)—T0	13.32		6.91	
Fasting insulin levels (µIU/mL)—T12	19.74		13.87	
Prolactin levels (ng/mL)—T0	10.90		7.86	
Prolactin levels (ng/mL)—T12	17.71		13.47	
PANSS total—T0	106.63		15.36	
PANSS total—T12	50.19		12.23	
PQ-LES-Q total—T0	22.63		4.48	
PQ-LES-Q total—T12	40.06		9.73	

NM, normal (extensive metabolizers); RFM, reduced-function metabolizers (IM/PM, intermediate/poor metabolizers); T0, baseline; T12, 12-month follow-up; BMI, body mass index. WT = Wild Type (the normal, fully functional allele). (*) = indicates that these are variant alleles of the CYP2D6 gene (WT/*4, WT/*3, WT/*41, *4).

**Table 3:** Antipsychotic medication, adverse effects, symptom severity and quality of life (comparatively for NM and RFM).

	NM (N = 27)		RFM (N = 20)		χ^2^ Test	Significance (*p*-Value)
	N	%	N	%		
Current antipsychotic medication						
Aripiprazole	13	48.2	3	15	7.59	0.06
Risperidone	3	11.1	6	30		
Olanzapine	8	29.6	10	50		
Quetiapine	3	11.1	1	5		
Adverse reactions to antipsychotic medication (T0–T12)						
BMI increase (>7%)						
No	20	74.1	8	40	5.53	0.02 *
Yes	7	25.9	12	60		
Fasting insulin levels > 25 µIU/mL						
No	23	85.2	14	70	1.58	0.28
Yes	4	14.8	6	30		
Hyperprolactinemia (>20 ng/mL in males and >25 ng/mL in females)						
No	24	88.9	13	65	3.92	0.04 *
Yes	3	11.1	7	35		
Sedation						
No	26	96.3	12	60	9.78	0.002 *
Yes	1	3.7	8	40		
Extrapyramidal symptoms						
No	25	92.6	10	50	10.96	0.001 *
Yes	2	7.4	10	50		
Sex hormone imbalances (dis-/amenorrhea, gynecomastia, galactorrhea)						
No	24	88.9	12	60	5.35	0.02 *
Yes	3	11.1	8	40		
	Mean	SD	Mean	SD	Mann–Whitney U test	Significance (*p*-value)
PANSS total—T0	103.93	13.66	110.30	17.06	U = 214, Z = −1.21	0.23
PANSS total—T12	42.51	7.63	60.55	9.25	U = 49.5, Z = −4.75	<0.0001 **
PQ-LES-Q total—T0	22.51	4.20	22.80	4.94	U = 261.5, Z = −0.18	0.85
PQ-LES-Q: general and emotional well-being sub-score—T0	5.74	1.19	5.50	1.35	U = 236.5, Z = −0.74	0.46
PQ-LES-Q: social functioning sub-score—T0	3.18	0.83	3.15	0.87	U = 258.5, Z = −0.26	0.79
PQ-LES-Q: independence and daily life sub-score—T0	4.88	1.12	5.30	1.26	U = 213, Z = −1.28	0.19
PQ-LES-Q: cognitive and physical functioning sub-score—T0	5.59	1.30	5.65	1.53	U = 269.5, Z = −0.01	0.99
PQ-LES-Q: leisure and life satisfaction sub-score—T0	3.11	0.93	3.20	1.00	U = 264.5, Z = −0.12	0.90
PQ-LES-Q total—T12	49.59	4.48	31.25	7.72	U = 23, Z = −5.33	<0.0001 **
PQ-LES-Q: general and emotional well-being sub-score—T12	11.96	1.76	8.10	2.07	U = 45, Z = −4.90	<0.0001 **
PQ-LES-Q: social functioning sub-score—T12	6.44	0.80	5.05	0.88	U = 70, Z = −4.79	<0.0001 **
PQ-LES-Q: independence and daily life sub-score—T12	9.96	1.28	6.60	1.84	U = 29, Z = −5.25	<0.0001 **
PQ-LES-Q: cognitive and physical functioning sub-score—T12	11.81	1.44	7.10	2.26	U = 33.5, Z = −5.21	<0.0001 **
PQ-LES-Q: leisure and life satisfaction sub-score—T12	6.41	0.57	4.40	1.46	U = 55.5, Z = −4.87	<0.0001 **

* *p* < 0.05, ** *p* < 0.001.

**Table 4:** Quality of life and adverse reactions to antipsychotic treatment.

	Mean Scores (SD), M-W Test Results (U, *p*-Value)						
PQ-LES-Q domains and total scores (T12)	BMI increase > 7%		Fasting insulin > 25 µIU/mL	Hyperprolactinemia	Sedation	Extrapyramidal symptoms	Sex hormone imbalances
General and emotional well-being score (GEWB)	No	11.61 (SD = 1.61)	10.83 (SD = 2.58)	11.0 (SD = 2.35)	10.97 (SD = 2.33)	11.17 (SD = 2.25)	11.05 (SD = 2.36)
	Yes:	8.42 (SD = 2.19)	8.40 (SD = 2.27)	7.80 (SD = 2.44)	7.55 (SD = 2.45)	7.83 (SD = 2.36)	7.90 (SD = 2.34)
	U = 84.5, *p* < 0.0001 **		U = 90.5, *p* = 0.013 *	U = 66.5, *p* = 0.002 *	U = 54.5, *p* = 0.001 *	U = 69.5, *p* = 0.001 *	U = 70.5, *p* = 0.001 *
Social functioning (SF)	No	6.25 (SD = 0.92)	6.00 (SD = 1.00)	6.08 (SD = 0.92)	6.13 (SD = 1.84)	6.14 (SD = 0.91)	6.08 (SD = 0.93)
	Yes	5.26 (SD = 1.04)	5.30 (SD = 1.25)	5.00 (SD = 1.24)	4.66 (SD = 1.22)	5.00 (SD = 1.12)	5.09 (SD = 1.22)
	U = 147.5, *p* = 0.004 *		U = 139, *p* = 0.182	U = 98, *p* = 0.012 *	U = 57.5, *p* = 0.001 *	U = 94, *p* = 0.002 *	U = 114, *p* = 0.019 *
Independence and daily life (IDL)	No	9.21 (SD = 1.89)	8.83 (SD = 2.16)	9.05 (SD = 1.91)	9.18 (SD = 1.72)	9.31 (SD = 1.67)	9.11 (SD = 1.90)
	Yes	7.52 (SD = 2.45)	7.40 (SD = 2.41)	6.60 (SD = 2.54)	5.77 (SD = 2.33)	6.25 (SD = 2.30)	6.63 (SD = 2.41)
	U = 174.5, *p* = 0.044 *		U = 118.5, *p* = 0.08	U = 85, *p* = 0.008 *	U = 40.5, *p* < 0.0001 **	U = 58.5, *p* < 0.0001 **	U = 84.5, *p* = 0.004 *
Cognitive and physical functioning (CPF)	No	10.75 (SD = 2.47)	10.32 (SD = 2.82)	10.48 (SD = 2.58)	10.76 (SD = 2.30)	10.82 (SD = 2.52)	10.50 (SD = 2.62)
	Yes	8.42 (SD = 3.16)	7.90 (SD = 2.84)	7.30 (SD = 3.09)	5.77 (SD = 1.92)	6.83 (SD = 2.08)	7.54 (SD = 3.04)
	U = 156.5, *p* = 0.015 *		U = 100.5, *p* = 0.025 *	U = 80.5, *p* = 0.005 *	U = 18.5, *p* < 0.0001 **	U = 51.5, *p* < 0.0001 **	U = 91, *p* = 0.006 *
Leisure and life satisfaction (LLS)	No	6.07 (SD = 0.89)	5.78 (SD = 1.31)	5.91 (SD = 1.08)	5.97 (SD = 0.99)	6.05 (SD = 0.90)	5.91 (SD = 1.10)
	Yes	4.78 (SD = 1.75)	4.70 (SD = 1.63)	4.20 (SD = 1.81)	3.77 (SD = 1.71)	4.08 (SD = 1.72)	4.36 (SD = 1.80)
	U = 155.5, *p* = 0.012 *		U = 108, *p* = 0.035 *	U = 80.5, *p* = 0.004 *	U = 49, *p* = 0.001 *	U = 72.5, *p* < 0.0001 **	U = 95.5, *p* = 0.007 *
PQ-LES-Q total score	No	43.89 (SD = 7.38)	41.78 (SD = 9.08)	42.54 (SD = 7.92)	43.02 (SD = 7.31)	43.51 (SD = 7.31)	42.66 (SD = 8.00)
	Yes	34.42 (SD = 10.20)	33.70 (SD = 9.86)	30.90 (SD = 10.70)	27.55 (SD = 8.93)	30.00 (SD = 9.08)	31.54 (SD = 10.37)
	U = 130.5, *p* = 0.003 *		U = 96, *p* = 0.020 *	U = 73, *p* = 0.004 *	U = 36, *p* < 0.0001 **	U = 52, *p* < 0.0001 **	U = 79.5, *p* = 0.003 *

* *p* < 0.05, ** *p* < 0.001.

**Table 5:** Coefficients, confidence intervals, and collinearity statistics for the regression model.

Coefficients ^a^										
Model		Unstandardized Coefficients		Standardized Coefficients	t	Sig.	95.0% Confidence Interval for B		Collinearity Statistics	
		B	Std. Error	Beta			Lower Bound	Upper Bound	Tolerance	VIF
1	(Constant)	50.731	3.623		14.003	0.000	43.404	58.059		
	PANSS total (T12)	−0.044	0.087	−0.056	−0.509	0.613	−0.220	0.131	0.345	2.898
	**Hyperprolactinemia**	**−4.473**	**1.707**	**−0.190**	**−2.621**	**0.012**	−7.926	−1.021	0.781	1.281
	**Sedation**	**−4.731**	**2.092**	**−0.193**	**−2.262**	**0.029**	−8.961	−0.500	0.562	1.778
	**Extrapyramidal symptoms**	**−5.472**	**1.748**	**−0.248**	**−3.131**	**0.003**	−9.007	−1.937	0.656	1.524
	BMI increase (>7%)	−2.972	1.577	−0.151	−1.885	0.067	−6.163	0.218	0.636	1.572
	**Metabolizer status (NM vs. RFM)**	**−7.995**	**2.120**	**−** **0** **.410**	**−3.771**	**0.001**	−12.283	−3.707	0.347	2.884
	Fasting insulin levels over 25 µIU/mL	−2.770	1.643	−0.118	−1.686	0.100	−6.092	0.553	0.843	1.186

^a^ Dependent variable: PQ-LES-Q total score (T12).

## Data Availability

The participants in this study did not provide written informed consent for the public release of their data. Given the sensitive nature of the research, supporting data cannot be provided.
